# Enhanced Protective Effect of the Combination of *Uncaria* and *Semen Raphani* on Vascular Endothelium in Spontaneously Hypertensive Rats

**DOI:** 10.1155/2015/358352

**Published:** 2015-10-11

**Authors:** Yun-lun Li, Yue-Hua Jiang, Chuan-Hua Yang, Jing-Chang Sun, Miao-Miao Wang, Wen-Qing Yang

**Affiliations:** ^1^Affiliated Hospital of Shandong University of Traditional Chinese Medicine, Jinan, Shandong 250011, China; ^2^Shandong University of Traditional Chinese Medicine, Jinan, Shandong 250355, China

## Abstract

Endothelial dysfunction and low-grade inflammation are closely associated with hypertension and other cardiovascular diseases. The combination of *Uncaria (U)* and *Semen Raphani (R)* is common in traditional Chinese medicine for the treatment of hypertension and heart diseases. We aimed to investigate the therapeutic effect of the combination of *Uncaria* and *Semen Raphani* on spontaneously hypertensive rats (SHRs), and valsartan was used as a positive control. In the present study, all extracts decreased systolic pressure, diastolic pressure, and mean arterial pressure. *U* alone showed antihypertensive efficacy and effectively decreased CECs count, while *R* alone showed efficacy in relieving inflammatory level. The combination of *U* and *R* showed enhanced effectiveness at lowering activated CECs and improving endothelial integrity of thoracic aorta and mesenteric artery and normalized the level of plasma biomarkers of endothelial damage. The combination of *U* and *R* decreased the mRNA level of VCAM-1, Sel-L, TFPI, and Sel-P, while it elevated mRNA expression of FGF-1 and THBD of the thoracic aorta, which may be, at least in part, involved in the mechanism of protective effect on hypertensive endothelial injury.

## 1. Introduction

Hypertension is a very common disease and an important risk factor in cardiovascular events, which are characterized by functional and structural vascular abnormalities. Vascular endothelium plays a fundamental role in modulating vascular tone and structure, and the endothelial dysfunction and resulting structural changes may be responsible for the adverse outcomes of hypertension [1]. Well-maintained endothelial function and integrity is of importance in numerous conditions, such as hypertension and inflammatory and cardiovascular disease and their risk factors [2, 3]. The damage of vascular endothelium leads to hypercoagulation, vascular inflammation, and an imbalance of oxidative and antioxidative systems. These pathological changes increase the risk of cardiovascular events and one of the pathological changes of hypertension is to convert mural endothelial cells into circulating endothelial cells (CECs). The amount of peripheral CECs correlates to disease intensity and functions as a valuable damage marker, and these cells are thought to originate from the sloughing-off of the vessel wall, following some form of pathological insult [4]. Increased CECs number in the blood is the product of a disease process that irreversibly damages the endothelium [5].

*Uncaria* (U) is a rubiaceous plant growing in South China. Total* Uncaria* alkaloids are the alcohol extract of the stems of* Uncaria*.* Rhynchophylline* and* Isorhynchophylline* are main pharmacological components in total* Uncaria *alkaloids. We know that* Rhynchophylline* and* Isorhynchophylline* have pharmacological effects similar to calcium channel antagonist, blood pressure lowering, antimyocardial structure, and antivascular smooth muscle cell and fibroblast proliferation [6, 7].* Semen Raphani* (R) is the mature seed of radish, helping digestive, antibacterial, and antiexotoxin effects.* Sinapine thiocyanate* is the main effective ingredient of* Semen Raphani*. In recent years, the focus of TCM research on this herb has been on its vascular dilation and antihypertensive functions. One of the advantages of TCM is the combination of several compatible herbs which may demonstrate dramatic efficacy.

What we are interested in is whether there is enhanced efficacy combining* Uncaria* and* Semen Raphani* to treat hypertensive disease. In the present study, we aimed to investigate the therapeutic effect of the combination of* Uncaria* and* Semen Raphani* on SHR, through the assessment of blood pressure, heart rate, the degree of endothelial damage, and overall inflammatory level, and then explored the possible pharmacological mechanism preliminarily by microarray analysis and quantitative real-time PCR.

## 2. Materials and Methods

### 2.1. Drugs and Reagents

*Total Uncaria alkaloids* and soluble* Semen Raphani alkaloid*, the two extracts, both of 50% purity, were provided by Pharmacological Department, Shandong University of Traditional Chinese Medicine. The content of* total Uncaria alkaloids* was determined by the acid dye staining method, and content of* Rhynchophylline* was determined by high performance liquid chromatography (HPLC). The fingerprint of* total Uncaria alkaloids* was established by HPLC and the content of* Rhynchophylline* accounted for more than 5.5% of* total Uncaria alkaloids*. The HPLC method was employed to ensure that the content of sinapine thiocyanate accounted for more than 42.53% in the soluble* Semen Raphani alkaloids*. After the dose screening experiments, the extracts were dissolved in saline before use and kept at 4°C. The final concentration of* total Uncaria alkaloids* solution is 3.853 mg/mL, and that of* Semen Raphani alkaloids *solutionis4.623 mg/mL. Valsartan was purchased from Beijing Novartis Pharma Ltd., Lot # x1417, dissolved in saline to a concentration of 1.335 mg/mL, and kept at 4°C.

Rat lymphocyte separation medium was purchased from Solarbio (Beijing, China, Lot # 20120516); FACS lysing solution was from BD (USA, Lot # 34543); Monensin Solution, Cell Stimulation Cocktail (500X), Anti-Rat CD54 (ICAM-1) PerCP-eFluor 710, Anti-Rat CD3PE, and TNF-*α* were from eBioscience (USA, Lot # E00020-1630, E13495-102, E14752-101, E00983-1634, and E00877-321); the improved RPMI-1640 culture medium was from Tianzhu Biochemical Products Co., Ltd. (Beijing, China, Lot # NYB0813); mouse serum was from Ding Guo Technology Co. Ltd. (Beijing, Lot # 23U00130); Triton X-100 was from Solarbio (Beijing, China, Cat. # T8200); CD31 (TLD-3A12) FITC and rabbit monoclonal antibody [EPR3208] to CD146 (ab75769) were from Abcam (UK, Lot # GR79311-3); goat anti-rabbit IgG-PerCP (sc-45090) and CD62P (P-selectin) [CTB201] PerCP were from Santa Cruz Biotechnology (USA, Lot # A3009).

### 2.2. Animals

200 male spontaneously hypertensive rats (SHRs), VAH/SPF level, 5-week-old, 94–120 g, and 40 age-matched normotensive Wistar Kyoto (WKY) rats were purchased from Beijing Weitong Lihua Experimental Animal Technology Co., Ltd. (certificate: SCXK (Beijing) 20120001). SHRs were randomly divided into 5 groups and intragastrically administrated for 6 days per week for the duration of 8 weeks: valsartan group (13.350 mg valsartan/kg body weight/d),* total Uncaria alkaloids* group (U group, 38.525 mg* total Uncaria alkaloids*/kg body weight/d), soluble* Semen Raphani alkaloids* group (R group, 46.230 mg soluble* Semen Raphani alkaloids*/kg body weight/d), 5 : 6 component compatibility of* total Uncaria alkaloids* group and soluble* Semen Raphani alkaloids* group (U-R group, combination of 38.525 mg* total Uncaria alkaloids* and 46.230 mg soluble* Semen Raphani alkaloids*/kg body weight/d), and hypertension model rats group. 20 SHRs and 20 WKY rats which were intragastrically given the same volume of saline for the same duration were used as control. The animals, housed under a 12 : 12 light-dark cycle, were allowed food and water* ad libitum* with normal salt intake. Ethical approval for the project was granted by the Faculty of Medicine & Health Sciences Ethics Committee for Animal Research, Affiliated Hospital of Shandong University of Traditional Chinese Medicine.

### 2.3. Detection of Blood Pressure and Heart Rate

Systolic pressure, diastolic pressure, mean arterial pressure, and heart rate were detected daily by the noninvasive rat tail method. Rats were collected and put in the ALC-HTP animal system, heated to dilate rat tail artery, and data were measured with ALC-NIBP noninvasive blood pressure analysis system [8]. All rats were measured 3 times in parallel, and data was shown as mean ± SD.

### 2.4. Ultrastructural Observation of Vascular Endothelium

Eight SHRs of each group were sacrificed at the end of the 1st, 2nd, 4th, 6th, and 8th week by overdose anesthesia with sodium pentobarbital (60 mg/kg, i.p.). After blood was collected by venipuncture, the thoracic aorta and mesenteric artery were taken immediately. The thoracic aorta was cut into two parts, one part for endothelial morphology observation by scanning electron microscope (SEM) and the other part for gene chip analysis and quantitive real-time PCR assay. For endothelial morphology observation, the thoracic aorta and mesenteric artery were isolated gently, washed, and fixed in 2.5% glutaraldehyde solution and then observed under SEM.

### 2.5. Measurement of the Number of Peripheral Circulating Endothelial Cells (CECs)

CECs in the peripheral blood can be considered an indicator of endothelial injury and vascular integrity [3]. CD31 is a constitutive marker expressed on endothelial cells (EC). CD146 is the key cell surface antigen on quantifying blood-borne endothelial cells.

The number of peripheral circulating endothelial cells was measured by indirect FACS-fluorescence labeled antibody by flow cytometry. Mononuclear cells were isolated from 2 mL hemolysin-treated EDTA-anticoagulant peripheral blood by centrifugation. The isolated mononuclear cells were labeled with CD146-PerCP 20 *μ*L, CD3-PE 0.25 *μ*g, and CD31-FITC 10 *μ*L and then detected by flow cytometry. CD3^−^CD31^+^CD146^+^ cells were regarded as peripheral circulating endothelial cells [5, 9]. The absolute value of WBC was counted with hemocytometer under microscopy. The amount of circulating endothelial cells was calculated by multiplying the absolute value of WBC by the percentage of peripheral circulating endothelial cells in WBC.

### 2.6. Observation of the Activity of Circulating Endothelial Cells

ICAM-1 (CD54) and P-selectin (CD62P) were taken as markers of CECs with activity and adhesive ability. The mean fluorescence intensity of CD54 and CD62P of CD3^−^CD31^+^ cells was detected by flow cytometry. Mononuclear cells were isolated from 1 mL lymphocyte separation medium treated with EDTA-anticoagulant peripheral blood by centrifugation. After being blocked with 20 *μ*L mouse serum, cells were triple-stained with anti-CD3-PE, anti-CD31-FITC, and anti-CD54-PerCP or with anti-CD3-PE, anti-CD31-FITC, and CD62P-PerCP. Fluorescence intensity was determined in a FACSCalibur flow cytometer.

### 2.7. Determination of the Level of Biomarkers of Endothelial Damage in Plasma

The contents of plasma nitric oxide (NO), endothelin-1 (ET-1), P-selectin (P-S), von Willebrand factor (vWF), intercellular adhesion molecule-1 (ICAM-1), and vascular cellular adhesion molecule-1 (VCAM-1) were assayed using ELISA kits (R&D Systems, USA, Lot # 20120718). All assays were performed according to the manufacturer's instructions. Wells were developed with tetramethylbenzidine in the dark and the well absorbance was measured at 450 nm. The protein content was quantified against a standard curve calibrated with known amounts of bovine serum albumin. All samples were assayed in triplicate and measurements were expressed as mean ± SD.

### 2.8. Assessment of TNF-*α* Secretion of T Cells Subsets in Spleen

Inflammation is one of the factors closely related to hypertension. The content of TNF-*α* in plasma associated with the spleen can reflect overall inflammatory state. The content of plasma TNF-*α* was assayed using ELISA kit (R&D Systems, USA, Lot # 20150320). The spleen was dissected under sterile conditions and ground gently through 200-mesh strainers. Thereafter, cells were collected by centrifugation and the sediment was resuspended in PBS. After T cell subsets were collected using lymphocytes separation medium, they were adjusted to a concentration of 5 × 10^6^ cells/mL and then stimulated with PMA plus ionomycin and monensin, for 4 hours. After that, cells were collected and fixed with 4% polyformaldehyde solution. Fc receptors on the cell surface were blocked with mouse serums, and cells were stained with anti-TNF-*α*-FITC antibodies; then the fluorescence intensity of the cells was detected in a FACSCalibur flow cytometer. All the data was analyzed with the software FACSDiva Version 6.13.

### 2.9. Microarray Analysis

In order to explore the mechanism of the protective effect of the combination of* Uncaria* and* Semen Raphani* on vascular endothelium, RT-PCR chip for biological function of endothelium was performed to observe mRNA expression of the thoracic aorta [10]. The total RNA of the thoracic aorta was isolated using the TRIzol method and then reverse-transcribed into cDNA with the PrimeScript RT reagent kit (RR047A, TaKaRa, Dalian, China). Following quantitative detection, the cDNA was hybridized in an Affymetrix Hybridization Oven 640, with subsequent elution inside a clean workstation (GeneChip Fluidics Station 450, Affymetrix). The chips were then scanned with a Gene Array Scanner 3000 7G (Affymetrix) to trace the detection signal. A GeneSpring GX 7.3.1 was used for the gene expression analysis and transcripts with low expression levels (<25% of the median gene expression value) and frequently missed hybridizations (>2 absent flags among the samples) were not analyzed further. Transcripts that showed a >2-fold expression reduction in tissue from drug-treatment groups relative to SHR groups were extracted. The transcript list contained 84 transcripts. Both the test group and control group included three biological replicates [11].

### 2.10. Quantitative Real-Time PCR

According to the results of the microarray analysis, key genes (fibroblast growth factor-1 (FGF-1), L-selectin (Sel-L), P-selectin (Sel-P), tissue factor pathway inhibitor (TFPI), thrombomodulin (THBD), and vascular cell adhesion molecule-1 (VCAM-1)) were chosen for quantitative real-time PCR to verify the gene expression results. The specific forward/reverse primer sequences (Sangon Biotech, Shanghai, China) were as follows: FGF-1 (5′-ACAGCAGCAGGAATGCATTGAG-3′/5′-AACTGTCGATGGTGCGTTCAAG-3′), Sel-L (5′-CCCTGAGCTGGGTACCATGAA-3′/5′-GCTGCTAGAGGCATGCACTGA-3′), TFPI (5′-CAGCAACAACTTTGAGACCTTGGA-3′/5′-GCGCTTTGGTAGCCTGAGGA-3′), THBD (5′- ACATATCTGAGACGGATGGATGGAA-3′/5′-TGGGACTACAAATGGCAAACACA-3′), VCAM-1 (5′-CGGTCATGGTCAAGTGTTTG-3′/5′-GAGATCCAGGGG AGA TGT CA-3′), and *β*-actin (5′-CGTTGACATCCGTAAAGA-3′/5′-AGCCACCAATCCACACAG-3′). Total RNA of vascular endothelium was isolated using the TRIzol method and reverse-transcribed into cDNA with the PrimeScript RT reagent kit. Real-time PCR was performed with a SYBR Premix Ex Taq kit (RR420A, TaKaRa, Dalian, China). The reaction mixture contained SYBR Green Premix Ex Taq, cDNA, and the forward/reverse primers. Reaction mixture and amplification conditions were maintained according to the manufacturer's instructions. Each RNA sample was tested in triplicate, and the threshold cycle values were normalized to *β*-actin and were presented as the mean ± SD. The relative gene expressions (fold change) of WKY rats, SHRs, valsartan group, U group, R group, and U-R group were calculated with the 2^−ΔΔCT^ method [12, 13].

### 2.11. Statistics

Statistical analyses were performed using SPSS 17.0. Values were presented as mean ± SEM. Unless stated otherwise, statistical comparisons were made using two-factor analysis of variance (ANOVA). *P* values of less than 0.05 were considered significant.

## 3. Results

### 3.1. Blood Pressure and Heart Rate

**Figure 1:**
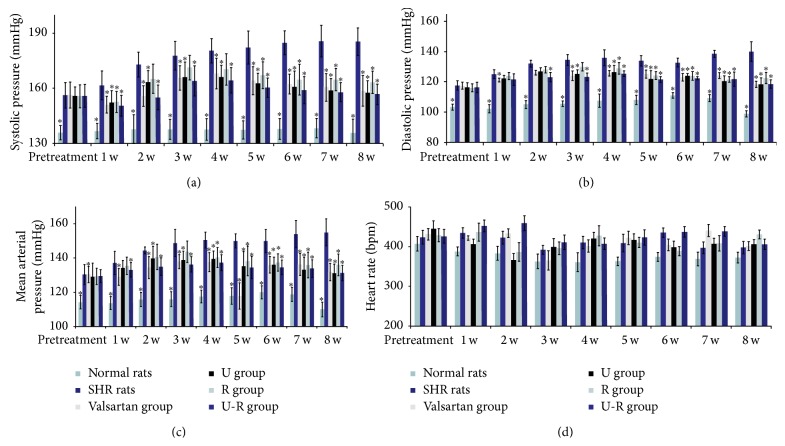
The changes of blood pressure and heart rate were detected by noninvasive rat tail method. Systolic pressure (a), diastolic pressure (b), and mean arterial pressure (c) of SHRs kept increasing gradually during the 8-week study. In the period of the first 4 weeks of drug treatment, systolic pressure, diastolic pressure, and mean arterial pressure of SHRs still increased and then decreased significantly. Heart rate (d) of SHRs changed slightly but there was no significance between groups. ^*∗*^ *P* < 0.05 versus SHRs.

Blood pressure of untreated SHRs kept increasing gradually during the study process and then began to decrease after being treated with drugs for a week (*P* < 0.05). The antihypertensive effect reached the highest point at the end of the 8 weeks, significantly different compared with that of untreated SHRs (*P* < 0.05). Systolic blood pressure decreased by about 30 mmHg in valsartan group, U group, and U-R group, while that in R group was less significant (20 mmHg) at the end of the 8-week treatment (Figure 1(a)). Diastolic pressure decreased by about 20 mmHg in all treatment groups (Figure 1(b)). Mean arterial pressure decreased by 19–24 mmHg in treatment groups at the end of the 8-week treatment (Figure 1(c)). Heart rates of the rats slightly decreased with advancing age, but there was no significant difference between groups during the 8-week study process (Figure 1(d)).

### 3.2. Endothelial Morphology Change

**Figure 2:**
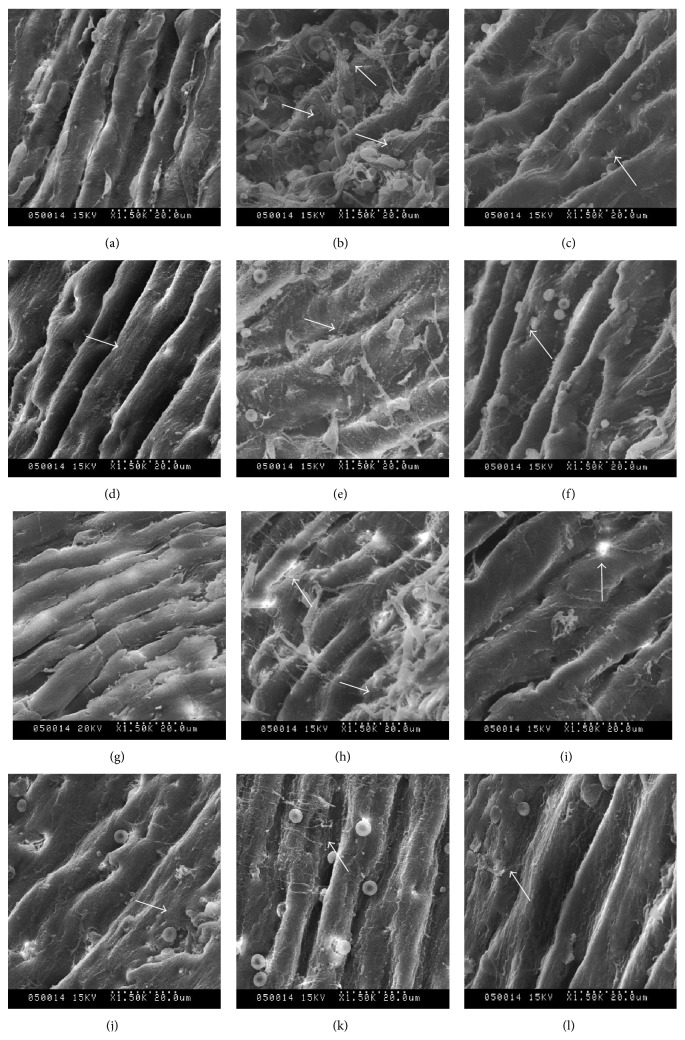
Endothelial morphologies of thoracic aorta and mesenteric artery were observed by scanning electron microscope after 8 weeks of drug treatment. (a)–(f) Endothelial morphology of thoracic aorta; (g)–(l) endothelial morphology of mesenteric artery. ((a) and (g)) WKY rats; ((b) and (h)) SHRs; ((c) and (i)) valsartan group; ((d) and (j)) U (*Uncaria*) group; ((e) and (k)) R (*Semen Raphani*) group; ((f) and (l)) U-R (effective components of* Uncaria* and* Semen Raphani*) group. The arrows point to the endothelial damage and the change of endothelial morphology.

The SEM study directly revealed the morphological change of vascular endothelium directly as shown in Figure 2; both large elastic arteries (thoracic aorta) and resistance vessels (mesenteric artery) were damaged seriously in SHRs. The vascular cord of WKY rats was arranged in neat rows, maintaining structural integrity and intercellular connection integrity, and mucosa was smooth, with no obvious fibre or plaque attached. The vascular endothelium of SHRs had shed significantly and aggregated, the cable was partly disordered, and cell connection was lost, with a “hole” or “honeycomb” shaped endometrium and with attachments adhering to the membrane. Endometrial integrity and shedding state of endothelial cells improved notably after drug treatment. U alone showed a significant protective effect on endothelium, which was better in U-R combination. The improvement effects of the drugs were U-R > valsartan > U > R. The best improvement groups were the valsartan group and U-R group.

### 3.3. The Amount and Activity of Peripheral Circulating Endothelial Cells

**Figure 3:**
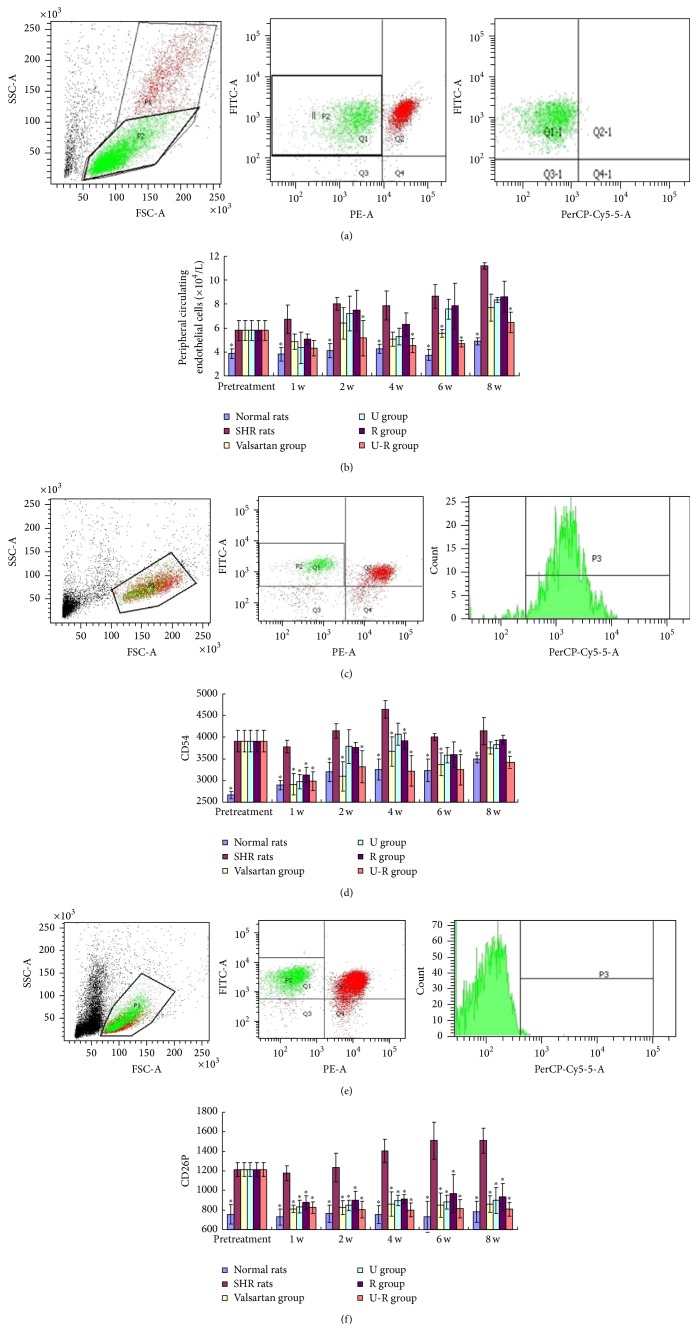
The amount and activity of peripheral CECs by flow cytometry. Identification of CD3^−^CD31^+^CD146^+^ cells as peripheral circulating endothelial cells (CECs) in the mononuclear cells by flow cytometry ((a) and (b)). Observation of activated CECs by CD3^−^CD31^+^CD54^+^ ((c) and (d)) and CD3^−^CD31^+^CD62P^+^ ((e) and (f)) by flow cytometry. ^*∗*^ *P* < 0.05 versus SHRs.

Compared with the WKY rats, CECs number of SHRs increased significantly and kept rising with advancing age (*P* < 0.05). When treated with* total Uncaria alkaloids* or soluble* Semen Raphani alkaloids*, respectively, the count of CECs decreased at different degrees (U group: 25%; R group: 23%) during 8 weeks (*P* > 0.05). The amount of CECs was decreased notably (*P* < 0.05; 37% at 2 weeks' end; 42% at 8 weeks' end) after administration of U-R for 2 weeks. The combination of U-R demonstrated the most powerful effect on decreasing the amount of CECs (Figures 3(a) and 3(b)).

Compared with the WKY rats, CD54 and CD62P expression on CECs of SHRs was increased significantly (*P* < 0.05). When treated with drugs, CD54 expression on CECs decreased in different degrees over the 8 weeks. U-R group showed the best efficacy (decreased by 20% at the end of the second week and 15.7% at the end of the eighth week), while the efficacy of other groups was not stable (Figures 3(c) and 3(d)). All groups showed a significantly low level of CD62P expression in CECs (*P* < 0.05), suggesting that all drugs had beneficial effects on decreasing activity of CECs (Figures 3(e) and 3(f)).

### 3.4. The Change of the Level of Biomarkers of Endothelial Damage in Plasma

**Figure 4:**
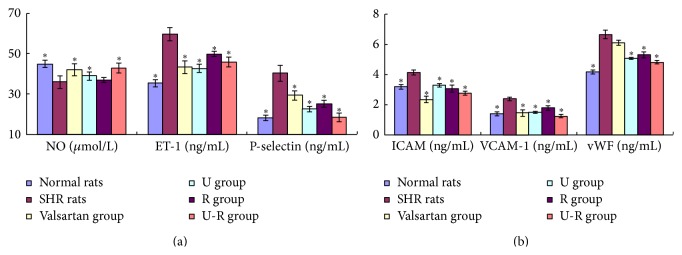
Assessment of the level of biomarkers of endothelial damage in plasma. The plasma level of NO, vWF, ET-1, ICAM-1, VCAM-1, and P-S was determined by ELISA to assess the degree of endothelial damage. ^*∗*^ *P* < 0.05 versus SHRs.

The plasma level of NO decreased; meanwhile, vWF, ET-1, ICAM-1, VCAM-1, and P-S increased significantly (*P* < 0.05) in SHR compared with the WKY rats. The level of all the biomarkers of endothelial damage normalized in different degrees with the treatments (Figure 4). Valsartan demonstrated excellent efficacy on regulation of ET-1, ICAM-1, and VCAM-1; U showed good efficacy in regulation of ET-1, vWF, VCAM-1, and P-S; R demonstrated good efficacy in regulation of vWF and P-S, while U-R had excellent efficacy in regulation of all the factors.

### 3.5. Assessment of the Overall Inflammatory Level

**Figure 5:**
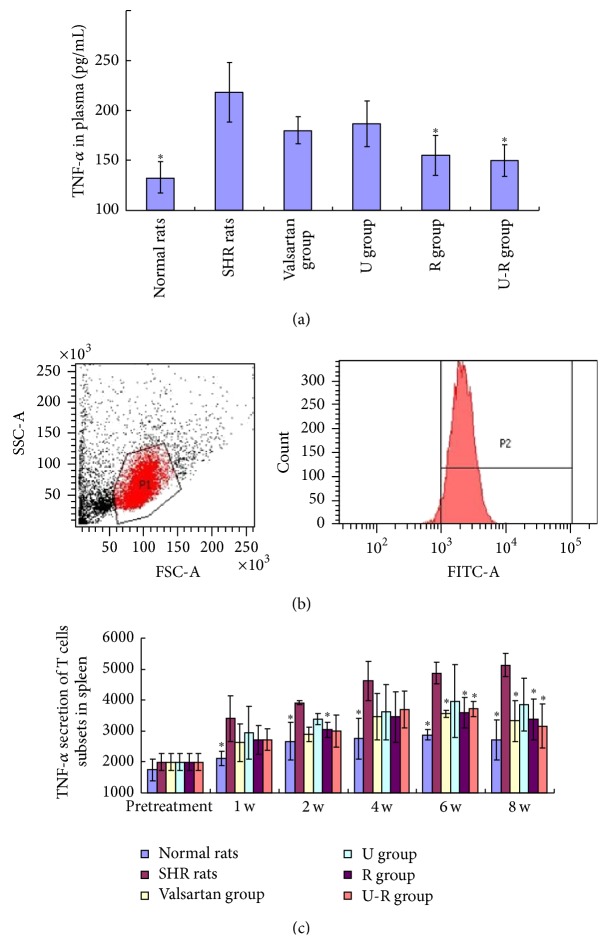
Assessment of the overall inflammatory level. The level of plasma TNF-*α* (a) was measured by ELISA. TNF-*α* secretion in spleen (b) was measured by flow cytometry. The level of plasma TNF-*α* and the mean fluorescence intensity of TNF-*α* on SHRs were much higher than those of WKY rats (*P* < 0.05). TNF-*α* was decreased after drug treatment, except in U group (*P* < 0.05). ^*∗*^ *P* < 0.05 versus SHRs.

The plasma level of TNF-*α* was increased (*P* < 0.05) in SHR compared with the WKY rats. The plasma level of TNF-*α* decreased in different degrees after drug administration (Figure 5(a)). The mean fluorescence intensity of TNF-*α* in T cell subsets of SHR's spleens was much higher than that of WKY rats (*P* < 0.05). TNF-*α* decreased to varying degrees after treatment, especially in R and U-R groups (*P* < 0.05). We speculated that this was one of the mechanisms of U-R of protecting vascular endothelium against hypertension (Figures 5(b) and 5(c)).

### 3.6. Expression of Key Genes

**Figure 6:**
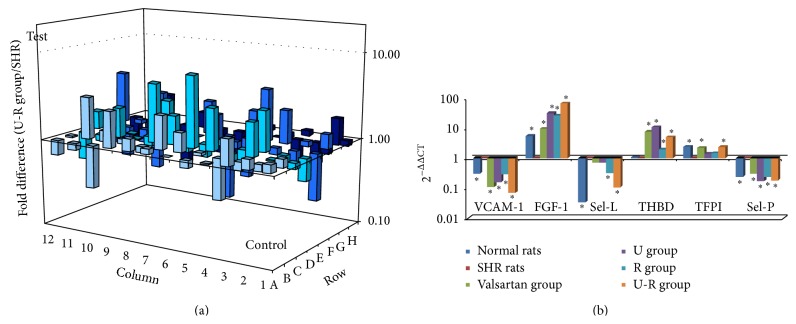
Changes of gene expression of the antithrombotic actions related genes. The pharmacological mechanism of U-R was speculated by microarray analysis (a), and the microarray results were verified and antithrombotic action related genes were determined by and quantitive RT-PCR (b). ^*∗*^ *P* < 0.05 versus SHRs.

Changes of gene expression in microarray analysis were listed in Table 1. QRT-PCR was then performed to confirm the microarray findings. Consistent with the gene chip data, the mRNA expression of VCAM-1, Sel-L, Sel-P, and TFPI was elevated and that of FGF-1 and THBD was decreased in vascular endothelium of SHRs. After drug treatment, the mRNA levels of these cytokines were normalized in different degrees (Figure 6).

## 4. Discussion

Hypertension, to some extent, is a vascular disease. Alteration of the vascular endothelium is a primary event in the pathogenesis of vascular diseases, such as atherosclerosis and systemic hypertension [14]. Hypertension and vascular endothelial dysfunction are a reciprocal causation. The abnormal hemodynamics and the change of shear force of hypertension may cause endothelial dysfunction, which is one of the initiating factors of endothelial injury. The injury of vascular endothelial cells often leads to the development of hypertension. Damaged endothelial cells tend to detach from the vessel wall, leaving a thrombogenic and proinflammatory subendothelial surface, and simultaneously release many active substances, such as thrombin, 5 HT, and endothelin, resulting in the disordered regulation of vascular tone and elevation of blood pressure [15]. Elevated blood pressure further aggravates the vascular endothelial injury and dysfunction. Therefore, it is vital to study the pathology of endothelial dysfunction and damage [16].

In the present study, we observed the vascular endothelial injury process of SHRs to speculate the pathophysiology in hypertension patients, as well as U-R's targets and pharmacological processes. The combined use of U and R effectively decreased systolic pressure, diastolic pressure, and mean arterial pressure. The efficacy of combination of U-R was better than that of U or R alone, demonstrating similar efficacy to valsartan. U-R showed particularly prominent efficacy on lowering systolic blood pressure, which provided the premise for U-R as vascular protective agents. U-R improved endothelial morphology of both large elastic arteries (thoracic aorta) and resistance vessels (mesenteric artery). As the morphology of elastic arteries improved, which helped cushion the shear force, systolic pressure was lowered. Accompanied by the improved morphology of resistance vessel, the passive expansion capability of peripheral resistance vessels was increased and diastolic pressure was reduced. U-R increased the plasma level of NO and decreased the level of vWF, ET-1, ICAM-1, VCAM-1, and P-S, suggesting the recovery of vascular function. The improvement of endothelial integrity and elastic vascular and resistance vascular function may be attributed to the antihypertensive effect.

Vascular endothelial cells (ECs) provide a nonthrombogenic and nonadhesive surface in healthy subjects, but under pathologic conditions they become proadhesive and procoagulant [17]. In normal steady-state conditions, the amount of mature CECs in the bloodstream is very low, due to the fact that endothelial turnover is a very slow process in the absence of pathological stimuli and that nonviable CECs are likely rapidly cleared by the reticuloendothelial system. The level of peripheral CECs is expected to increase as a consequence of any type of damage to the vessel wall [18, 19]. In this study, the amount of CECs of SHRs was much higher than that of WKY rats. During the 8-week study, the peripheral CECs count of SHRs kept increasing, while that of WKY rats stayed stable. The count of CECs in treatment groups also increased but was much lower than that of SHRs, especially in valsartan and U-R groups. The combination of U-R demonstrated even better efficacy (41.8%) on lowering CECs count of valsartan (30.3%). Not only the amount but also the cell status of CECs has valuable significance [20]. We take ICAM-1 (CD54) and P-selectin (CD62P) as markers of activated CECs. Endothelial adhesion molecule CD54 (ICAM-1) mediates cellular adhesion and transcellular migration. CD62P is expressed appreciably in inflammatory conditions and participates in a variety of pathological changes [21]. All drugs showed beneficial efficacy on decreasing CD62P expression. But only the combination of* Uncaria* and* Semen Raphani* could effectively decrease CD54 to approximate level in WKY rats, which may effectively reduce the risk of vascular injury and thrombosis.

Inflammation contributes to the pathophysiology of hypertension. We observed TNF-*α* level in both plasma and T cell subsets of SHR's spleens to assess overall inflammatory level [22]. TNF-*α* promotes inflammatory responses and activates the adhesion molecules, which leads to the injury of endothelial cells resulting in endothelium dysfunction, vasomotor strengthening, diastolic weakening, and systemic small artery spasm; thus, blood pressure is elevated [23]. Both* Semen Raphani* alone and combination of U-R showed good efficacy in relieving inflammation. Effectively reducing the low-grade inflammation may be one of the pharmacological ways in which* Semen Raphani* is antihypertensive. U alone showed antihypertensive efficacy and effectively decreased CECs count, while R alone showed efficacy in relieving inflammatory level. The combination of U and R proved the enhanced endothelial protective effects.

According to the results of microarray and qRT-PCR, we believe that the expression and regulation of antithrombotic actions and adhesion related genes are closely related to endothelial dysfunction. A common function of the endothelium is to maintain blood in a fluid state and to limit clot formation when there is a breach in the integrity of the vascular wall. On the anticoagulant side, ECs express tissue factor pathway inhibitor (TFPI), heparan, thrombomodulin, endothelial protein C receptor (EPCR), tissue-type plasminogen activator (t-PA), ecto-ADPase, prostacyclin, and nitric oxide. On the procoagulant side, ECs synthesize tissue factor, plasminogen activator inhibitor- (PAI-) 1, von Willebrand factor (vWF), and protease activated receptors [24]. Regulation of coagulation action and inhibition of the inflammatory cytokine and adhesion molecules may alleviate endothelial injury and relieve vascular wall inflammation, to slow the progress of hypertension. The results of the present study suggested that combination of U-R decreased the mRNA level of VCAM-1, Sel-L, TFPI, and Sel-P, while elevating mRNA expression of FGF-1 and THBD of the thoracic aorta. The regulation of extracts of U-R on antithrombotic actions and adhesion related genes may contribute to the mechanism of the regulation on hypertension and endothelium injury.

## 5. Conclusions

The combination of extracts of* Uncaria* and* Semen Raphani* (*total Uncaria alkaloids* and soluble* Semen Raphani alkaloid*) demonstrated good antihypertensive effect and vascular endothelium protective effect. The possible mechanism of this protective effect on vascular endothelium may be attributed to the regulation of antithrombotic actions related genes (FGF-1, Sel-L, Sel-P, TFPI, THBD, and VCAM-1) and relieving the overall low-grade inflammation.

## Figures and Tables

**Table 1:** Changes in gene expression (fold Change, 2^−ΔΔCt^).

	WKY rats/SHRs	Valsartan group/SHRs	U group/SHRs	R group/SHRs	U-R group/SHRs
VCAM-1	0.55	0.41	0.60	0.69	0.29
FGF-1	6.00	3.27	2.82	2.97	6.64
Sel-L	0.53	0.23	0.24	0.30	0.35
THBD	5.89	3.33	3.34	3.46	3.96
TFPI	0.26	0.32	0.23	0.21	0.31
Sel-P	0.24	0.30	0.17	0.24	0.18
